# Sorption and Mobility of Charged Organic Compounds: How to Confront and Overcome Limitations in Their Assessment

**DOI:** 10.1021/acs.est.2c00570

**Published:** 2022-03-30

**Authors:** Gabriel Sigmund, Hans Peter H. Arp, Benedikt M. Aumeier, Thomas D. Bucheli, Benny Chefetz, Wei Chen, Steven T. J. Droge, Satoshi Endo, Beate I. Escher, Sarah E. Hale, Thilo Hofmann, Joseph Pignatello, Thorsten Reemtsma, Torsten C. Schmidt, Carina D. Schönsee, Martin Scheringer

**Affiliations:** † Department of Environmental Geosciences, Centre for Microbiology and Environmental Systems Science, University of Vienna, 1090 Wien, Austria; ‡ Norwegian Geotechnical Institute (NGI), P.O. Box 3930 Ullevaal Stadion, N-0806 Oslo, Norway; § Norwegian University of Science and Technology (NTNU), NO-7491 Trondheim, Norway; ∥ RWTH Aachen University, Institute of Environmental Engineering, Mies-van-der-Rohe Straße 1, 52074 Aachen, Germany; ⊥ Environmental Analytics, Agroscope, 8046 Zürich, Switzerland; # Department of Soil and Water Sciences, Institute of Environmental Sciences; Faculty of Agriculture, Food and Environment, The Hebrew University of Jerusalem, P.O. Box 12, Rehovot 7610001, Israel; ¶ College of Environmental Science and Engineering, Ministry of Education Key Laboratory of Pollution Processes and Environmental Criteria, Tianjin Key Laboratory of Environmental Remediation and Pollution Control, Nankai University, Tianjin 300350, P. R. China; ∇ Wageningen Environmental Research, Wageningen University and Research, P.O. Box 47, 6700AA, Wageningen, Netherlands; ☆ Health and Environmental Risk Division, National Institute for Environmental Studies (NIES), Onogawa 16-2, 305-8506 Tsukuba, Ibaraki Japan; ■ Department of Cell Toxicology, Helmholtz Centre for Environmental Research − UFZ, Permoser Strasse 15, DE-04318 Leipzig, Germany; ∂ Environmental Toxicology, Center for Applied Geoscience, Eberhard Karls University Tübingen, Schnarrenbergstr. 94-96, DE-72076 Tübingen, Germany; ∞ Department of Environmental Sciences, The Connecticut Agricultural Experiment Station, New Haven; 123 Huntington St., New Haven, Connecticut 06504-1106, United States; ⬢ Department of Analytical Chemistry, Helmholtz Centre for Environmental Research − UFZ, Permoserstrasse 15, 04318 Leipzig, Germany; √. Institute for Analytical Chemistry, University of Leipzig, Linnéstrasse 3, 04103 Leipzig, Germany; Å Instrumental Analytical Chemistry and Centre for Water and Environmental Research (ZWU), University of Duisburg-Essen, Universitätsstrasse 5, 45141 Essen, Germany; ⊗ RECETOX, Masaryk University, 625 00 Brno, Czech Republic; ● Institute of Biogeochemistry and Pollutant Dynamics, ETH Zürich, 8092 Zürich, Switzerland

**Keywords:** ionizable organic compound, anion, cation, zwitterion, sorption model, environmental risk assessment, contaminant fate

## Abstract

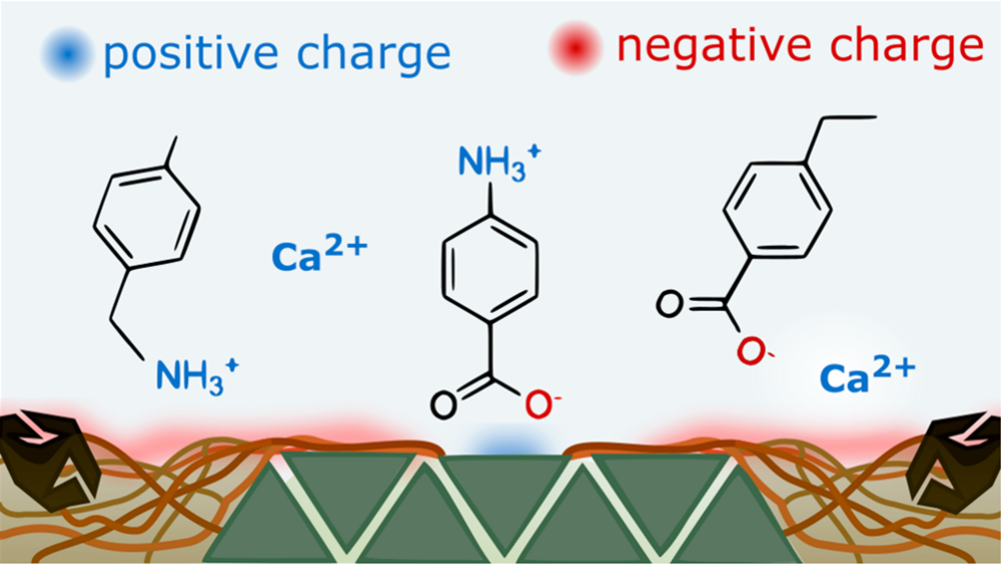

Permanently charged and ionizable organic compounds (IOC) are a large and diverse group of compounds belonging to many contaminant classes, including pharmaceuticals, pesticides, industrial chemicals, and natural toxins. Sorption and mobility of IOCs are distinctively different from those of neutral compounds. Due to electrostatic interactions with natural sorbents, existing concepts for describing neutral organic contaminant sorption, and by extension mobility, are inadequate for IOC. Predictive models developed for neutral compounds are based on octanol–water partitioning of compounds (*K*_ow_) and organic-carbon content of soil/sediment, which is used to normalize sorption measurements (*K*_OC_). We revisit those concepts and their translation to IOC (*D*_ow_ and *D*_OC_) and discuss compound and soil properties determining sorption of IOC under water saturated conditions. Highlighting possible complementary and/or alternative approaches to better assess IOC mobility, we discuss implications on their regulation and risk assessment. The development of better models for IOC mobility needs consistent and reliable sorption measurements at well-defined chemical conditions in natural porewater, better IOC-, as well as sorbent characterization. Such models should be complemented by monitoring data from the natural environment. The state of knowledge presented here may guide urgently needed future investigations in this field for researchers, engineers, and regulators.

## Abstract 2

We argue for the development of new concepts to describe mobility of charged organic compounds and present starting points.

For regulators, engineers, and researchers, the mobility of contaminants is crucial for assessing their potential to contaminate groundwater and surface waters. The mobility of an organic compound is generally inversely related to its tendency to sorb. Widely used approaches to assess sorption were developed for neutral compounds but are inadequate to describe the complex behavior of permanently charged and ionizable organic compounds (IOC). Common examples of IOC are weak acids and bases that have a pH-dependent fraction of species with a negative or positive charge, respectively, due to (de)protonation. Some compounds are permanently charged (ionic) under environmental conditions and/or exist in a zwitterionic form with both positive and negative charges in the same structure. Numerous contaminants of concern are IOC, including many pharmaceuticals, pesticides, industrial chemicals, such as dyes and polymer building blocks, as well as most per- and polyfluoroalkyl substances, and natural toxins.

Compounds that are (partially) charged at environmental pH make up more than half of all substances recently categorized as priority “persistent, mobile, and toxic” (PMT) or “very persistent, very mobile” (vPvM) substances. These compounds pose a threat to clean and safe drinking water if emitted in substantial volumes, due to their high mobility, persistence, and limited removability from water.^1−3^ In addition, approximately 48% of all compounds registered under Europe’s REACH regulation are (partially) charged at environmentally relevant pH (4–9).^4^ A recent screening for persistent, mobile (PM), and vPvM compounds in surface water underlines the importance of IOC, as 85% of the identified compounds were expected to be charged at environmental pH.^5^ What distinguishes IOC from well-studied neutral organic compounds is that their sorption behavior, and consequently, their mobility in the environment, depends, often dramatically, on the local pH, water hardness, and mineral composition of soils or sediments. Therein, IOC sorption, but also bioaccumulation,^6^ and ecotoxicity^7^ strongly differ between uncharged neutral, negatively charged, positively charged, and zwitterionic species.

Sorption affinity can be expressed as the solid–water equilibrium distribution coefficient *K*_d_, which is the ratio of chemical concentration in the solid phase (*C*_s_, μg/kg) to that in the aqueous phase (*C*_aq_, μg/L) at equilibrium:
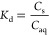
1For neutral organic compounds, it has been established since the 1980s that soil/sediment organic matter (SOM) is the key sorptive phase (sorbent).^8,9^ To ease comparison of sorption data between different sorbents, it is common practice to normalize measured *K*_*d*_ values to the fraction of organic carbon in soil or sediment (*f*_OC_), resulting in *K*_OC_ values (L/kg_OC_), that allow for a more generalizable quantification of organic compound sorption:^10^
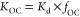
2

While the *K*_OC_ for a given compound is not a universal constant and can vary with the structure and composition of SOM, variation of *K*_OC_ in common soil and sediment organic matter is typically within a factor of 2,^11^ or in the worst case an order of magnitude for neutral organic chemicals.^12^ However, *K*_OC_ can increase by several orders of magnitude if the SOM includes highly condensed aromatic fractions of pyrogenic material (“black carbon”).^13^ Nevertheless, *K*_OC_ is commonly used to assess contaminant mobility in regulatory frameworks such as the European Biocide regulation,^14^ and the Food and Agriculture Organization of the United Nations guideline on soil contamination.^15^ As experimental *K*_d_ and *K*_OC_ values are not always available, octanol–water partitioning based approaches are commonly used to estimate these parameters for screening purposes.

Here, we maintain that the widely used octanol–water partitioning- and *K*_OC_-based approaches are not well applicable for assessing sorption and mobility of IOC. We discuss compound and soil properties driving sorption of IOC, highlight limitations of current models, and discuss possible complementary and/or alternative approaches to better assess IOC mobility for researchers, engineers, and regulators.

## Octanol–Water Partitioning

Following pioneering work by Karickhoff et al. in 1979^9^ for sediments, quantitative relationships between the *K*_OC_ and the octanol–water partition coefficient (*K*_ow_) obtained in independent experiments have been widely applied in sorption and mobility assessments of neutral hydrophobic compounds:^16^
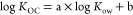
3where *K*_ow_ is the ratio of concentrations in the (water-saturated) octanol and (octanol-saturated) water at equilibrium, and *a* and *b* are regression parameters. The application of *K*_ow_ as a proxy for *K*_OC_ to assess organic compound sorption assumes that partitioning into the bulk SOM phase is the predominant sorption process, and that octanol is a good surrogate for SOM, which as we will discuss later, for IOC it is not.

Since the neutral, charged, and (if relevant) zwitterionic species of IOC partition differently into octanol, in these cases *K*_ow_ is replaced with an operational partitioning ratio called *D*_ow_. *D*_ow_ is the concentration ratio of the sum of all species in octanol (*C*_0_) to the sum of all species in water (*C*_w_) at equilibrium and at a given pH and ionic composition:
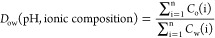
4

Generally, partition of a charged compound into octanol requires partition of an accompanying counterion to maintain electroneutrality in solution. Therefore, the extent to which a charged species partitions from water into octanol depends on the concentration and type of available counterions in the aqueous phase.^17,18^ If it is assumed that partitioning of the charged species is negligible compared to the neutral species, the calculation of *D*_ow_ is simplified to^19^
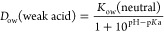
5
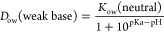
6

However, if the charged species do interact with soil constituents as explored in the next sections, the approach is inadequate to estimate sorption and mobility of IOC. Moreover, eqs 5 and 6 cannot be used for permanently charged compounds such as quaternary ammonium cations, where *K*_ow_ (neutral) does not exist. Additionally, hydrophobic domains in other parts of the IOC, charge delocalization over many atoms in the IOC (e.g., dinoseb, pentachlorophenoxide^17^), as well as hydrophobic organic counterions, can facilitate partitioning of an IOC into octanol as net-neutral ion pairs. Lastly, surfactant-like IOCs with a hydrophobic tail (e.g., many per- and polyfluoroalkyl substances) can form emulsions at high concentrations, which could affect their partitioning between organic matrices (octanol/SOM). Models to estimate *D*_ow_ are generally not capable of adequately accounting for these factors, resulting in erroneous *D*_ow_ estimates. This is especially true for cations and zwitterions, where models such as the Estimation Programs Interface (EPI) Suite^20^ do not yield meaningful estimates. For example, the EPI Suite by default assigns very low *D*_ow_ values (log *D*_ow_ = −6) to compounds with quaternary nitrogen structures, but ignores the ionized moiety in other compounds and treats them as if they were neutral.^21^ Even more importantly, as we will explore in the next sections, no matter how *D*_ow_ is determined, *D*_ow_ is not suitable for modeling IOC sorption when the charged species substantially affects sorption.

## Octanol–Water Partitioning Is Not Suitable for Describing IOC mobility

**Figure 1:**
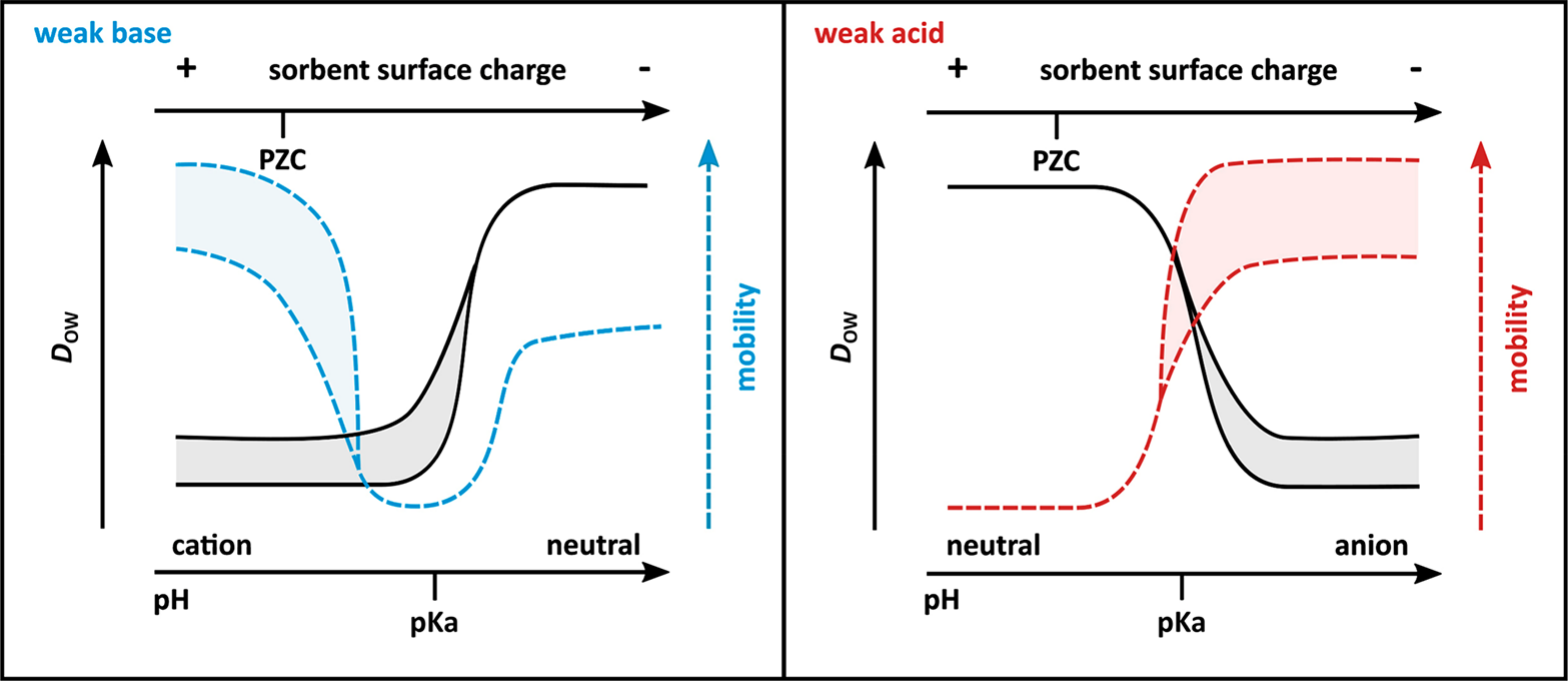
Mobility of IOC in soils and sediments depends not only on hydrophobicity, but is additionally affected by the surface charge of soil constituents, pore water chemistry, and IOC speciation. PZC = sorbent point of zero charge; above this pH overall surface charge is negative, D_ow_ = water-chemistry dependent octanol–water partitioning coefficient, p*K*_a_ = IOC dissociation constant. Black solid lines and colored dashed lines represent hydrophobicity and mobility, respectively. The colored ranges represent the influence of counterion concentration.

The application of *K*_ow_ as a proxy for *K*_OC_ to assess organic compound sorption and mobility assumes that partitioning into the SOM phase is the only/dominant sorption process. Models based on *K*_ow_ or *D*_ow_ do not consider that increasing pH results in increasing negative charge density in soil,^22^ as explained later. This negative charge repels organic anions and attracts organic cations, which *D*_ow_ cannot reflect, as shown in Figure 1.

Weak bases which form cations at pH < p*K*_a_ of the corresponding acid, experience electrostatic repulsion at very low pH, which increases their mobility, followed by a minimal mobility due to electrostatic attraction toward negatively charged mineral and SOM moieties with increasing pH, and finally an intermediate mobility at pH ≫ p*K*_a_, where the neutral species is predominant.^23^ In contrast, for weak acids, *D*_ow_ would be high and mobility would be correspondingly low at pH ≪ p*K*_a_, where the compound exists predominantly in the neutral form. As the pH transitions through the p*K*_a_, *D*_ow_ is expected to decrease and mobility to increase as the compound is converted to the anionic form which is repulsed by negatively charged soil moieties.

The type and concentration of naturally occurring (counter)ions can modulate IOC sorption and mobility, as illustrated by the dashed lines in Figure 1. Importantly, the (counter)ion-dependent change in *D*_ow_ does not cover the changes on the sorbent side brought about by the presence of counterions. For example, for cations, *D*_ow_ increases with higher salinity because of the increased concentration of counterions that aid formation of ion pairs.^17,18^ However, in real soils or sediments the higher concentration of cations would compete for sorption sites and thus actually decrease sorption of cationic compounds.^24,25^ By contrast, (counter)ions could increase sorption for anionic compounds by decreasing electrostatic repulsion from negatively charged moieties.

## Octanol is not a Suitable Surrogate for SOM

The free energy of sorption (Δ*G*_sorp_), which is linearly related to the logarithm of *K*_d_, can be expressed as the sum of the contributions from net driving forces for removal of the solute from water and placing it in association with the solid. These driving forces include: van der Waals forces of dispersion and induction (Δ*G*^vdW^); polar forces including dipole–dipole, charge-dipole, and hydrogen (H)-bonding (Δ*G*^polar^); Coulomb interactions between full charges (Δ*G*^coul^), and the hydrophobic effect (Δ*G*^hyd^). The hydrophobic effect, also referred to as cavity formation energy, results from the sum of forces that limit the solubility of molecules in water. Its underlying cause is the disruption of the cohesive energy of water due to the greater ordering of water molecules and the lower number of water–water H-bonds in the hydration shell of the nonpolar moiety compared to the bulk water phase.^26−28^

**Figure 2:**
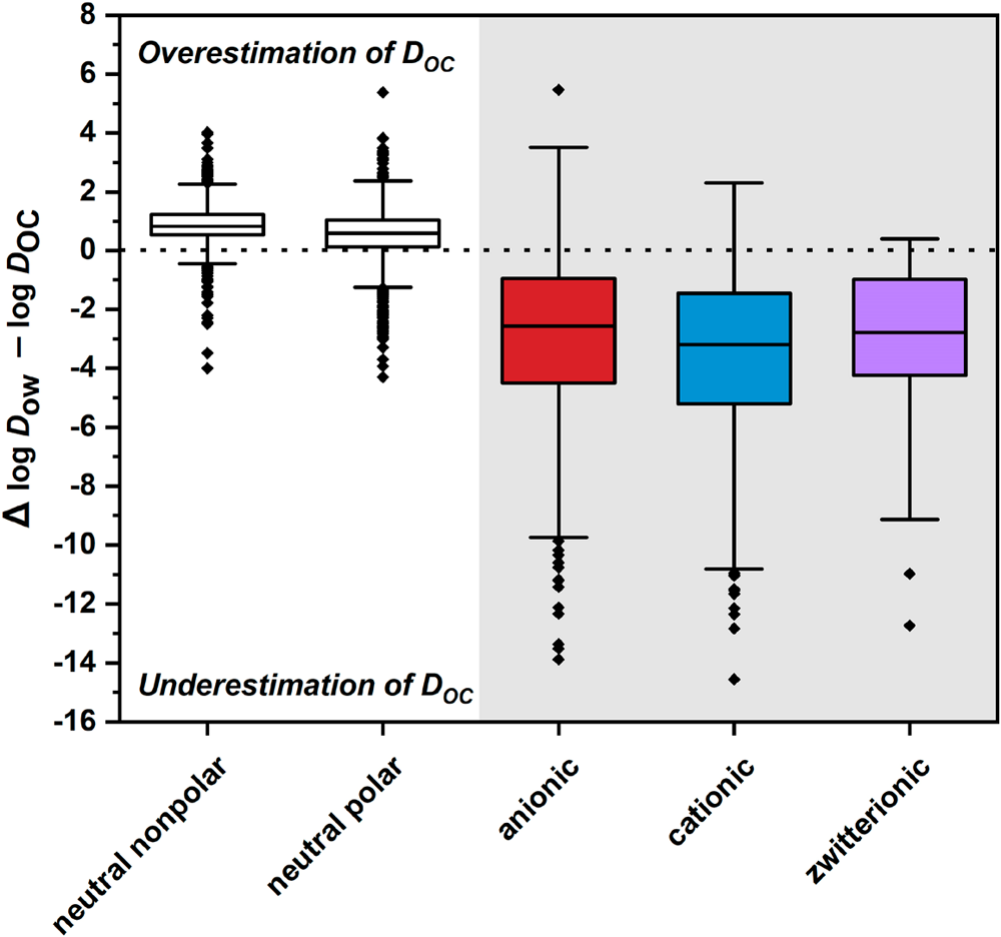
Differences (Δ) comparing lowest available *D*_ow_ in the pH range 4−9^19^ with measured *D*_OC_. *D* = *K* for neutral compounds. The dotted line at Δ = 0 indicates the point where *D*_ow_ = *D*_OC_. Charged species are highlighted in color. The extent of the boxplot relates to the uncertainty associated with predicting sorption from *K*_ow_/*D*_ow_ for a given compound. The middle line in the box corresponds to the median, the box to the 25% quantiles and the whiskers to the 1.5-fold interquartile range. *D*_ow_ being extremely lower than experimental *D*_OC_ is substantially influenced by the larger pH dependence of *D*_ow_ over this pH range, and Coulombic interactions with SOM not being considered in *D*_ow_. All boxplots are based on data presented in more detail by Arp et al.,^30^ which compiled experimental *K*_OC_, *K*_ow_, and p*K*_a_ data from the eChemPortal,^31^ and additional sources.^29,32^ Sample size: neutral nonpolar (*n* = 703), neutral polar (*n* = 1066), anionic (*n* = 488), cationic (*n* = 607), zwitterionic (*n* = 71).

Octanol is regarded an acceptable surrogate for SOM with respect to Δ*G*^hyd^ and Δ*G*^vdW^. Thus, as shown in Figure 2, the best estimations of *K*_OC_ from *K*_ow_ exist for neutral, nonpolar molecules, where on average the log *K*_OC_ is slightly smaller than log *K*_ow_.^9,29^ Octanol is less suitable with respect to Δ*G*^polar^ because octanol engages only in dipolar and ordinary (weak) H-bonding interactions of its aliphatic – OH group and misses many other polar interactions between sorbates and SOM. This is the reason why *K*_OC_*-K*_ow_ correlations are somewhat poorer for polar compared to apolar compounds.^29^ For IOC, where Δ*G*^coul^ is relevant, the pH-dependent “*D*_OC_“ has become a common parameter used instead of *K*_OC_.^3,19^ For IOC, octanol is even less suitable as a surrogate for SOM with respect to Δ*G*^coul^ because, unlike SOM, octanol contains no charged groups. Consequently, for *D*_ow_-derived *D*_*OC*_ estimations of IOC, errors substantially increase further and become meaningless. As shown in Figure 2, available *D*_ow_ values can be several orders of magnitude smaller than experimentally measured *D*_OC_ values for IOC, due to both Δ*G*^coul^ not being accounted for by *D*_ow_ and the pH dependence being substantially more sensitive for *D*_ow_ than *D*_OC_.

## IOC can Partake in a Variety of Interactions in Soil not Represented by Octanol

There are a number of sorption mechanisms of IOC in soil/sediment that are not captured at all by octanol-based models. Partitioning of organic compounds into octanol, whether they are ionized or not, is generally linear with solute concentration. While (ab)sorption of most neutral compounds into “soft” amorphous SOM phases is also close to linear, the same is not true for minerals and “hard” crystalline SOM phases (e.g., coal, black carbon), which can show moderate to strong nonlinearity of (ad)sorption with solute concentration.^10^ Here, the *K*_d_ generally decreases with increasing concentration, because adsorption sites are occupied preferentially in the order of the energy gain they enable, which varies. Deviation from linearity is more pronounced for organic anions and cations relative to neutral molecules, showing L- or H-type isotherms and additional sorbent-specific effects (e.g., for black carbon).^33,34^

**Figure 3:**
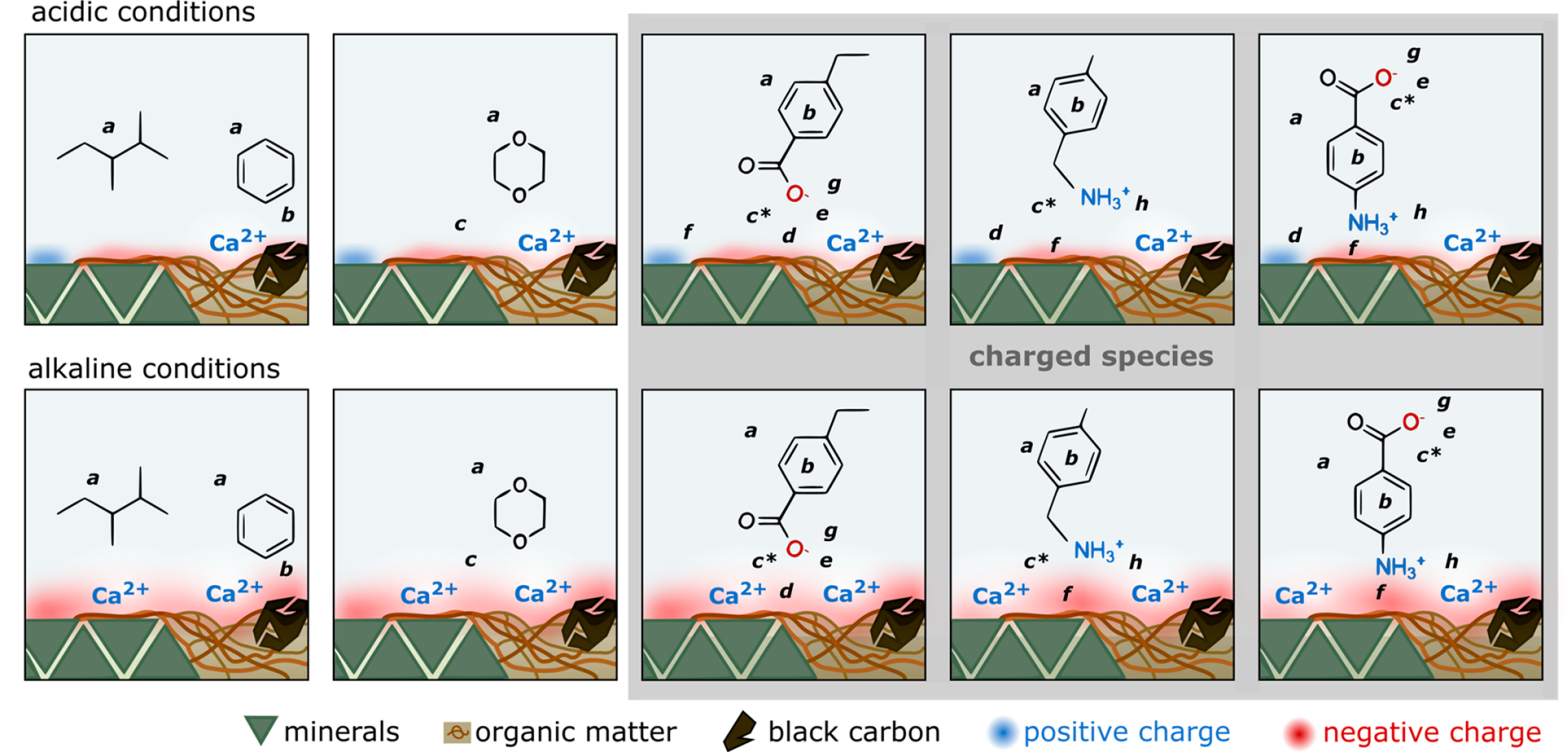
Key drivers and interactions for sorption of different groups of organic compounds under acidic conditions (top row) and alkaline conditions (bottom row). Compound groups with representative examples from left to right: neutral nonpolar compounds, neutral polar compounds, anionic compounds, cationic compounds, and zwitterionic compounds. Panels with charged species are highlighted in gray. Possible drivers and interactions: a = hydrophobic effect, b = π–π electron donor–acceptor interaction, c = H-bond, c* = charge assisted H-bond, d = electrostatic repulsion, e = cation bridging, *f* = electrostatic attraction, g = anion – π bond, h = cation – π bond.

As Illustrated in Figure 3, a number of interactions that do not occur for neutral compounds can occur for charged species (panels highlighted in gray in Figure 3). None of the following interactions are possible with octanol: Nonspecific electrostatic attraction or repulsion by charged moieties can direct the sorption of charged species (d,f in Figure 3), which can be described by the Donnan potential.^35^ Specific interactions of charged species with individual sorption sites widely differ among IOC, but often involve interactions between charged functional groups or aromatic structures in the IOC.^36,37^ The degree of aromatic condensation of SOM and black carbon can play an important role in the sorption of aromatic and heterocyclic compounds, which can interact via several types of π-electron donor–acceptor interactions (b,g, h in Figure 3).^38−41^ Weak acids and bases are capable of forming very strong, “charge-assisted” H-bonds (CAHB, c* in Figure 3) when acidic sites on SOM and black carbon have similar p*K*_a_ values as the IOC.^42^ The degree of hydration can also affect sorption site accessibility by crowding out solute molecules,^43^ or by disrupting SOM–SOM contact points within the solid phase.^44,45^

## SOM is not Always the Predominant Sorbent of IOC

Sorption models based on *K*_OC_/*D*_OC_ are conceptually not sufficient for capturing the full range of factors influencing IOC mobility in many soils and sediments. Such sorbents are complex mixtures of minerals, SOM, black carbon, colloids, and pore water containing dissolved organic matter (DOM) and dissolved inorganic ions, including anions such as Cl^–^, NO_3_^–^, H_2_PO_4_^–^/HPO_4_^2–^, SO_4_^2–^, and HCO_3_^–^/CO_3_^2–^, as well as cations such as Na^+^, K^+^, Ca^2+^ and Mg^2+^. IOC sorption to surfaces and nanometer-size pores of minerals and black carbon can be affected by all these substances.^36,46,47^

Most soil constituents, including SOM, black carbon, phyllosilicate minerals, and Mn oxides, exhibit an overall negative surface charge at pH of 4–9.^22^ The negative charge predominating on soil/sediment surfaces derives mainly from oxygen-containing functional groups that dissociate with increasing pH (e.g., carboxyl-, and hydroxyl groups). These functional groups determine the solid’s capacity to bind cations via cation exchange interactions, which can be quantified as the cation exchange capacity (CEC) at a given pH. SOM, black carbon, and clay minerals are especially high in CEC and are thus crucial to the mobility of cations.^48^ Sorption of organic cations to clay minerals depends on surface charge distribution, as well as type of exchangeable cations.^49^ Some organic anions (e.g., carboxylates, sulfonates) can also undergo surface bonding on mineral surfaces by ligand exchange with the underlying metal ions.^33^

On the other hand, only ∼7% of the numerous minerals in global soils have surfaces that are net positively charged at ambient pH, most importantly Fe-oxides and Al-oxides.^22^ Anion exchange can occur in the presence of these positively charged minerals. However, anion exchange capacity (AEC) is usually much smaller than CEC. As DOM and many types of colloids in the porewater are composed of negatively charged polyelectrolytes of different molecular sizes, which can compete with IOC anions for positively charged sites that are accessible to them. Thus, whatever AEC is inherent to soils or sediments is reduced by adsorption of DOM and/or aggregation with negatively charged minerals.

## Implications for Regulation and Risk Assessment

Regulatory criteria for contaminant mobility in soil are critically important to protect surface water, groundwater, and drinking water.^50^ The emphasis of mobility for risk assessment has recently been reinforced in the European Commissioǹs “Chemicals Strategy for Sustainability towards a Toxic Free Environment”, which states that mobility should be included in a wide number of activities related to chemical regulation, in order to reduce exposure to hazardous substances via groundwater, drinking water and other pristine water bodies.^51^

In 1989 Gustafson^52^ combined soil half-lives and *K*_OC_ values to estimate pesticide leachability. Today these two parameters are still used, as substances that degrade easily or sorb strongly are less prone to percolate to groundwater or pass bank filtration. For instance, the European regulatory framework for bioicides uses a *K*_OC_ of 500 L/kg_OC_ and soil half-life of 21 days as threshold values for groundwater risk assessment.^14^*K*_OC_ and *D*_OC_ threshold values for the mobility criteria for PMT and vPvM substances to be adopted by the European Classification, Labeling and Packaging (CLP) and REACH regulations are currently under discussion, and are expected to be finalized in 2022.^53^ As of now, the thresholds being investigated by the European Commission are a log *D*_OC_ < 3 within a pH range of 4–9 to be considered mobile, and substances with a log *D*_OC_ < 2 to be considered very mobile.^19,54^ Revised European chemical regulations that include PMT/vPvM substances could potentially mandate experimental *D*_OC_ assessments of all persistent substances in Europe, which is a key market for the chemical industry.

Currently, experimental *K*_d_ values, which would reflect the variety of possible soil (mineral) compositions and water–chemical conditions in the environment, are not widely available. Thus, estimated *D*_OC_ or *D*_ow_ values could be used as screening parameter to prioritize substances for experimental determination. As discussed previously, errors in the *D*_ow_-to-*D*_OC_ correlations for IOC can be substantial and are more pronounced for modeled than for experimental *D*_ow_ data.^19^ This renders the use of *D*_ow_ for risk assessment problematic. However, this does not invalidate the role of *K*_ow_ as a screening parameter for neutral nonpolar and neutral polar compounds, or arguably very large *D*_ow_ to screen for nonmobility of IOCs (considering *D*_ow_ are generally < *D*_OC_). *D*_ow_ is, however, not capable of substituting experimentally determined sorption parameters for IOC. For local mobility assessments, *D*_OC_ or even soil-specific *K*_d_ values need to be measured, due to substantial uncertainties in *D*_ow_ extrapolations. To aid the comparison of such values, soil mapping could be helpful, using databases from soil sciences and regulators.^55,56^ Still, local measurements are not always possible, and even if they were, they are impractical for inclusion in generalized chemical regulation.

## Moving Forward

In addition to simple relationships between sorption and *K*_ow_/*D*_ow_, more sophisticated quantitative structure–property relationships (QSPR) exist to estimate the sorption of neutral compounds to a vast number of sorbents.^57^ The appeal of these approaches is their capacity to yield mechanistic insights into sorption in dependence of compound properties (e.g., polarizability, H-bonding abilities). QSPR approaches based on such descriptors for charged species have been proposed.^58,59^ However, as the behavior of charged compounds strongly depends not only on pH, but also on the ionic composition in water, determining generalizable descriptors is not always straightforward. In addition, most QSPR approaches are developed for pure solvents or sorbents and fall short of describing complex mixtures of SOM, minerals, and black carbon which contain varying sorption sites and exhibit different CEC.

Mobility and sorption of IOC are more complex and variable than that of neutral compounds, as a larger number of factors can modulate their behavior. Most key interactions for charged compounds are not driven by hydrophobicity but rather by IOC speciation and sorbent surface charge, as well as the amount and composition of other ions in solution. Because of the complexity of IOC mobility, the emergence of a single and generalizable best-for-all parameter as alternative to experimentally determined *K*_OC_/*D*_OC_ values is unlikely. It is important to deduce from the discussion above, that for IOC, experimentally determined *K*_d_ for soil should not simply be converted to *D*_OC_ since multiple soil components contribute to overall IOC sorption and mobility. Until better approaches are developed, experimentally determined *K*_d_, and by extension *K*_OC_/*D*_OC_, values for diverse soil or sediment types are the only available parameters for initial sorption and mobility assessments for chemical regulation.

For cations, where electrostatic attraction to negatively charged surfaces often drives sorption, CEC normalized *K*_d_ values (*K*_CEC_) have been proposed as a complementary approach to the use of *K*_OC_.^46^ Sorption of organic cations to specific soil components (standardized SOM and Illite clay), have been compared to sorption to natural soils.^46^ This comparison found that sorption to the clay fraction had a negligible contribution to the *K*_d_ for an OC-enriched soil, whereas for a clayish soil the SOM sorption strongly underestimated the *K*_d_, which could be largely accounted for by including the Illite clay sorption affinity. For organocations, mobility estimates for a suite of soil types could thus be based on simple experimental measurements (in this example *f*_OC_, CEC_soil_, *K*_SOM_, *K*_clay_). In another study, maximum sorption capacity of black carbon for the dicationic herbicide paraquat was proportional to the square of the CEC of the black carbon, suggesting that the dication associated in a bidentate fashion with appropriately spaced negative sites on the sorbent.^43^ Thus, measurements of IOC sorption to pure soil constituents (SOM, clay minerals, black carbon) at specific pH and ionic strength conditions may offer a solid base for improved IOC mobility estimates. Although no one single “standard” SOM exists, a growing sorption data set on IOC has become available for Pahokee peat,^60,61^ and many processes such as influence of ionic strength, hardness, and pH dependency are relatively constant for other SOM types.^60^

In future approaches, *D*_OC_ could be complemented by pH-dependent *K*_CEC_ for cations, and extended to pH- and ionic-strength-dependent sorption measurements of key scenarios (e.g., a soil with a low OC content, a high CEC, and a low ionic strength would likely show large discrepancies between *D*_OC_ and *K*_CEC_). A similar approach could also be developed for anions. To close the gap between regulation and science, researchers may develop compound-group specific “realistic worst-case” scenarios that could be applied in risk assessment. For example, considering interactions in Figure 3, anions could be investigated at very high pH and low ionic strength, where electrostatic repulsion increases mobility and the available cations for charge shielding and cation bridging are minimized. By contrast, the mobility of cations could be measured at low pH and high ionic strength, where soils are partially positively charged, CEC is lowered, and inorganic cations can compete for sorption sites. A more detailed categorization of IOC would need to be developed for such an approach to account for complex molecules with multiple functional groups, as well as physical accessibility to sorption sites resulting from differences in sorbate conformation, sorbent geometry, and chemical structure (e.g., aromaticity).

Neural-network-based models combining compound and sorbent parameters could yield improved estimations for IOC sorption,^47^ and combined with sensitivity analysis may be a good starting point to identify key parameters for further model development. Ideally, in future approaches, molecular and geometrical properties of IOC will specify which interactions a given species can undergo and allow for categorization and prioritization of compound classes. This categorization could then result in a set of descriptors and/or probe compounds tailored to the compound class of interest. Based on these compound groups, tailored predictive models based on consistent sets of experimental data could be developed. These data should include sorption coefficients to a number of well characterized soil constituents (SOM, black carbon, clay minerals) as well as soils and sediments with varying compositions using high throughput experimental systems, as can be run using soil column chromatography approaches.^62^ Such approaches could also account for additional factors affecting IOC sorption, such as DOM and other compounds competing for sorption sites, as well as temperature, which can also alter IOC and sorbent functional group speciation.^63^ Field monitoring of potential contaminants under saturated conditions would be a valuable complementary approach to measuring sorption under well-defined conditions. Recent developments in analytical chemistry make it possible to measure a very wide range of IOC in environmental samples.^5,64^ These measurements may aid future model developments and allocation of IOC to substance classes with different environmental behavior.

Predictive approaches will continue to be necessary at least for preliminary assessment and screening purposes. To develop better models for IOC mobility, it is crucial to create consistent and reliable data sets with (i) well documented and correctly determined molecular properties including p*K*_a_ and *D*_ow_, (ii) well documented sorbent properties including organic carbon and black carbon content, mineral composition as well as pH dependent CEC, (iii) sorption data measured under different well-defined chemical conditions in water and soil (pH and ionic composition) under saturated conditions, and (iv) the consideration of additional complex interactions such as the air–water interface under unsaturated conditions which are important for a number of compound such as per- and polyfluoroalkyl substances.^65^ Predictive models aiming to improve risk assessment should integrate findings from monitoring studies for model calibration and validation, which can help to identify conceptual shortcomings and to expand the scope of a given model on a relevance and need basis.
