# Correction to “Sorption and Mobility of Charged Organic Compounds: How to Confront and Overcome Limitations in Their Assessment”

**DOI:** 10.1021/acs.est.2c05051

**Published:** 2022-07-20

**Authors:** Gabriel Sigmund, Hans Peter H. Arp, Benedikt M. Aumeier, Thomas D. Bucheli, Benny Chefetz, Wei Chen, Steven T. J. Droge, Satoshi Endo, Beate I. Escher, Sarah E. Hale, Thilo Hofmann, Joseph Pignatello, Thorsten Reemtsma, Torsten C. Schmidt, Carina D. Schönsee, Martin Scheringer

Unfortunately, due to a clerical error, the subscripts in eq 2 were mixed up in our feature on sorption and mobility of charged organic compounds.

The correct equation is
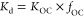
2Units for *K*_d_ are L/kg, units for *K*_OC_ are L/kg_OC_, and units for *f*_OC_ are g_OC_/g_soil_.

